# Aqueous extracts of *Portulaca oleracea* L. alleviate atopic dermatitis by restoring skin barrier function

**DOI:** 10.3389/fphar.2025.1591394

**Published:** 2025-06-05

**Authors:** Haobin Wei, Zhang Chen, Wenjie Lai, Wenxian Wang, Xiqing Bian, Limin Zhang, Xinzhi Li

**Affiliations:** ^1^ School of Pharmacy, Faculty of Medicine and Laboratory of Drug Discovery from Natural Resources and Industrialization, Taipa, Macao, China; ^2^ State Key Laboratory of Quality Research in Chinese Medicines, Macau University of Science and Technology, Taipa, Macao, China; ^3^ State Key Laboratory of Magnetic Resonance and Imaging, Innovation Academy of Precision Measurement Science and Technology, The Chinese Academy of Sciences, Wuhan, China

**Keywords:** purslane, aurantiamide acetate, epidermal barrier, cutaneous inflammation, JAK1 inhibition, filaggrin

## Abstract

**Background:**

*Portulaca oleracea* L*.* (PO) is an edible plant with a long medicinal history in traditional Chinese medicine for various inflammatory diseases, including skin disorders such as atopic dermatitis (AD). However, the anti-inflammatory effects and AD-alleviating mechanisms of PO remain unclear.

**Methods:**

PO aqueous extract was prepared from a water-soluble portion and then mixed with carbomer to obtain a hydrogel, which provided stable drug permeation and absorption in mouse skin. Amantadine acetate was identified as an abundant ingredient and further predicted to be a Janus kinase 1 (JAK1) inhibitor via molecular binding simulation. Mice with AD, established by repeated sensitization with 2,4-dinitrochlorobenzene (DNCB), were topically treated with PO hydrogel. Aurantiamide acetate was applied to HaCaT keratinocytes prior to inflammatory challenge in the presence or absence of JAK1-siRNA. Transcriptional and translational gene expressions associated with cutaneous inflammation or skin barriers were assessed by qPCR and Western blotting, respectively. Enzyme-linked immunosorbent assays were performed to detect immunoglobulin E and proinflammatory factors in skin tissue or serum. The phosphorylation of JAK1, signal transducer and activator of transcription (STAT)3, and STAT6 in keratinocytes and skin were analyzed by Western blotting.

**Results:**

In DNCB-sensitized mice, the PO hydrogel ameliorated skin lesions, lowered symptom scores, and reduced epidermal thickness by suppressing proinflammatory factor generation, oxidative stress, and the expression of CD4^+^. The PO hydrogel promoted the expression of caspase-14 and filaggrin, thereby helping restore skin barrier function in AD. The PO hydrogel and/or aurantiamide acetate inhibited the enzymatic activity of JAK1 and downstream (STAT)3/STAT6 signaling pathways *in vitro* and *in vivo*.

**Conclusion:**

PO significantly ameliorated skin lesions and restored epidermal barrier function in AD mice. This was achieved by suppressing JAK1 enzymatic activity and JAK1-mediated STAT signaling pathways.

## 1 Background

Atopic dermatitis (AD), a common chronic inflammatory, relapsing skin disease, is characterized by intense pruritus and eczematous lesions. AD is a major contributor to the global burden of skin diseases, with a prevalence of up to 20% in children and 10% in adults (Stander, 2021). The pathophysiology of AD involves a complex interplay between immune dysregulation, cutaneous inflammation, and epidermal barrier dysfunction (Bieber, 2022). Significant advancements have been made in the management of moderate-to-severe AD, including the use of dupilumab, which targets the interleukin (IL)-4 receptor subunit α; tralokinumab, which targets IL-13; secukinumab, which targets IL-17A; and various pan/dual-/selective-Janus kinase (JAK) inhibitors (Bieber, 2022).

At present, topical corticosteroids are still widely used as the first-line treatment for AD. However, improper use of steroids can lead to severe adverse reactions, such as cutaneous atrophy, vascular damage, telangiectasia, acne, and contact hypersensitivity reactions (Weidinger et al., 2018). In recent years, the development of selective JAK1 inhibitors has led to the emergence of novel topical treatments for AD (Simpson et al., 2020). However, these medications are associated with high costs and serious side effects in high-risk populations (Simpson et al., 2020), and very few options are available that can restore epidermal barrier function. Therefore, dermoprotective agents from ethnopharmacological origins may serve as an attractive option to mitigate cutaneous dysfunction with their proved efficacy, better safety profile, and cost-effectiveness. Traditional medicines and herbal preparations offer potential solutions to address this need.

*Portulaca oleracea* L. (PO), also known as purslane, is a widely distributed plant used for medicinal purposes and as a dietary resource. Traditionally, both fresh and dried plants can be administered for different purposes. Among various applications, PO extracts have been reported to benefit a range of skin disorders, such as wounds (Rashed et al., 2003) and ultraviolet damage (Oh et al., 2023). Both PO aqueous extract and ultrasound-assisted ethanol extract could alleviate clinical symptoms such as erythema, edema, dryness and ulceration in the back and ear in AD mouse model induced by 2,4-dinitrochlorobenzene (DNCB) (Lv et al., 2022). Additionally, fermented PO extract can dampen cutaneous inflammation in AD model mice via the nuclear factor (NF)-κB pathway (Zhao et al., 2023). However, the mechanisms and active ingredients of these extracts for AD management have not yet been deeply explored. Aurantiamide acetate, one of the most abundant alkaloids found in PO (Liang et al., 2014), exerts antioxidant and anti-inflammatory effects by blocking the NF-κB pathway (Zhou et al., 2017). The mechanistic role of aurantiamide acetate in preventing AD skin damage has not been investigated.

The present study aimed to prepare a carbomer hydrogel of PO aqueous extract and to investigate the effects of this hydrogel in an AD mouse model. The components of PO were analyzed via ultra high-performance liquid chromatography-quadrupole time-of-flight mass spectrometry (UHPLC-Q-TOF-MS), and aurantiamide acetate was predicted to be an active compound. Further studies demonstrated that both PO aqueous extracts and aurantiamide acetate suppress inflammation, orchestrate T helper cell-mediated immune responses, and restore the epidermal barrier by inhibiting JAK1 activity and downstream signal transducer and activator of transcription 3/6 (STAT3/6) signaling pathways.

## 2 Materials and methods

### 2.1 Preparation of carbomer hydrogel from PO aqueous extracts

**FIGURE 1:**
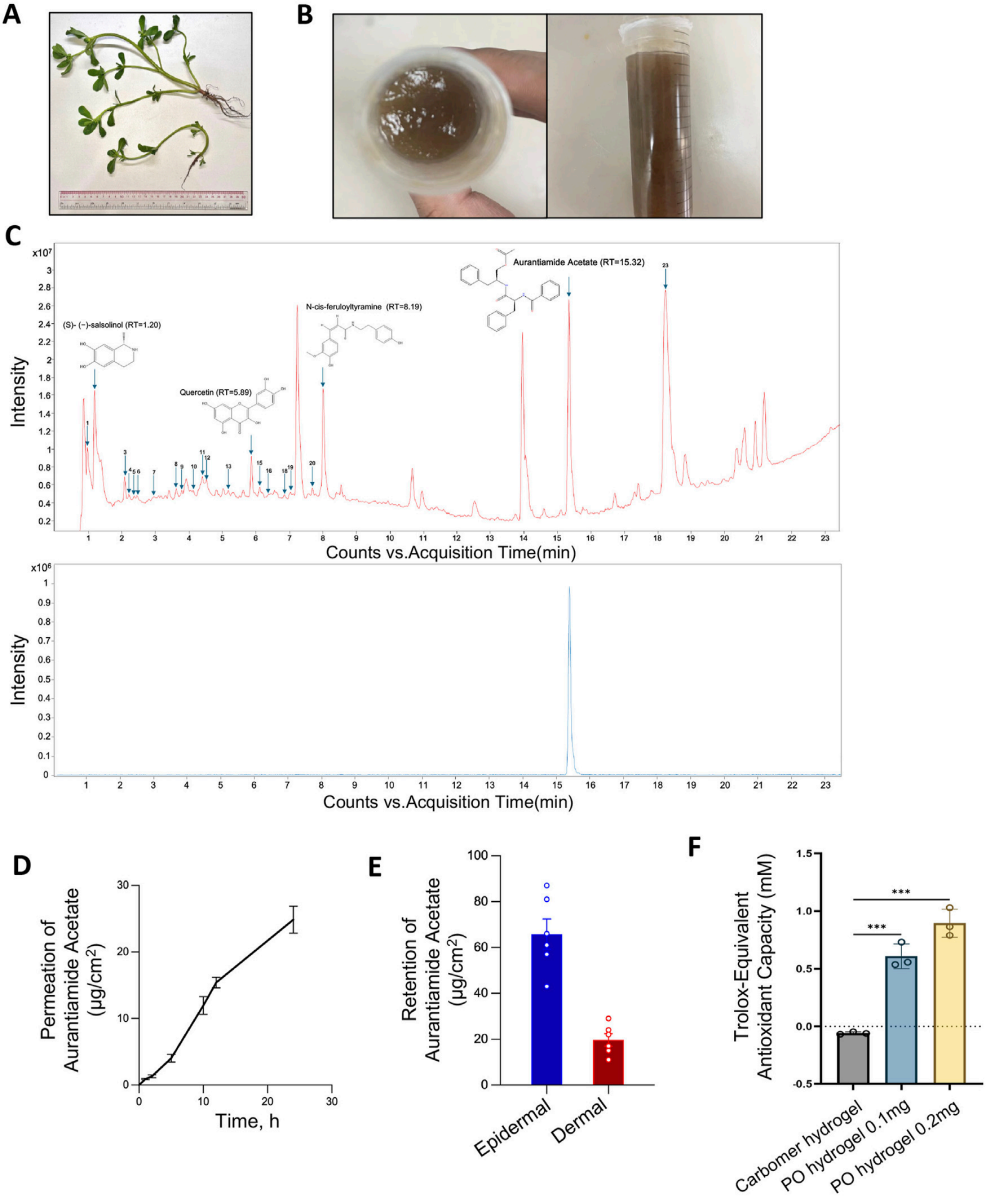
Preparation and characterization of the carbomer hydrogel of PO aqueous extract. **(A)** Fresh *Portulaca oleracea* L*.* **(B)** Prepared carbomer hydrogel of PO aqueous extract. **(C)** Chromatography of PO aqueous extracts by UHPLC-Q-TOF-MS/MS analysis in positive ion mode. The identified compounds are numbered in order on the basis of retention time. Upper panel, PO aqueous extracts; lower panel, reference compound aurantiamide acetate, with a retention peak at 15.32 min. Rt, retention time. **(D)** The cumulative permeation of aurantiamide acetate, one of the active ingredients in PO aqueous extract, was determined by UHPLC-Q-TOF-MS/MS in dissected rat skin at various time points after topical administration. n = 6. **(E)** Epidermal and dermal retention of aurantiamide acetate was determined by UHPLC-Q-TOF-MS/MS in dissected rat skin 24 h after administration. n = 6. **(F)** *In vitro* antioxidant ability of PO hydrogel. n = 3. The data are expressed as means ± SD.

Aerial parts, including stems and leaves of PO (Figure 1A), were purchased from Kuming City, Yunnan Province, China, and authenticated by the Faculty of Chinese Medicine, Macau University of Science and Technology. 2 kg of washed and air-dried fresh PO sample were sterilized by ultraviolet for 45 min. A juicer (Angelia 5500, Angel, United States) was used to extract 1300–1350 mL of juice from sterilized PO. The juice was then filtered through 5 layers of sterile gauze to obtain a light reddish PO aqueous extract that was further condensed to 500 mL.

For hydrogel preparation, carbomer 940 powder 10 g (Macklin, C832684, Shanghai, China) was dissolved in deionized water (w:v = 1:30) and the hydrogel was stabilized for 36 h. Glycerol 50 g, sodium p-hydroxybenzoate 20 g, disodium EDTA 40 g, and sodium bicarbonate 8 g were mixed in water and stirred for 5 min at room temperature. The hydrogel and suspension obtained in the initial two steps were then combined with 150 mL of PO aqueous extract for PO hydrogel or 150 mL of deionized water for carbomer hydrogel. With ultraspeed stirring (FSH-2A, Fangke Instrument, Changzhou, China) for 15 min, the sodium hydroxide solution (1 M) was added drop by drop to the solution to adjust the pH to 7. Finally, add water to make up to 1000 mL (Figure 1B).

### 2.2 Chemical characterization of PO aqueous extract via ultrahigh-performance liquid chromatography‒mass spectrometry (UHPLC‒Q-TOF‒MS/MS)

PO aqueous extract (10 µL) was extracted with 990 µL methanol in an ultrasonic bath (40 Hz, 300 W) for 30 min and then centrifuged at 10,500 × g for 10 min. An Agilent 1290 Infinity LC system (UHPLC, Santa Clara) and a binary pump with a Waters ACQUITY UPLC BEH C18 column (2.1 × 100 mm, 1.7 µm) were used for the separation of the mixtures. The column temperature was maintained at 30°C, and the autosampler was set at 8°C. The flow rate was 0.3 mLmin^−1^. Mobile phases A and B were 0.1% formic acid-containing water and 0.1% formic acid-containing ACN, respectively, and the gradient was set as follows: 0–28 min, 5%–95% B; 28–30 min, 95%–5% B. Mass spectrometry was conducted on an Agilent 6546 Q-TOF/MS system. The instrument was operated in positive (POS) ion mode. The MS parameters were set as follows: dry gas temperature at 300°C, dry gas flow at 11 Lmin^−1^, sheath gas temperature at 320°C, sheath gas flow at 11 Lmin^−1^, nebulizer pressure at 35 psig, capillary voltage at 3500 V, and nozzle voltage at 1000 V. The mass spectra were recorded across the range of m/z 100–1700 for derivatized samples. Aurantiamide acetate (MW = 444.5), a PO alkaloid purchased from ChemFaces (CFN98937, Wuhan, China) (purity ≥98%), was used as a standard compound to analyze the prepared PO extract and transdermal absorption (see below). Serial dilutions at 0.625, 1.25, 2.5, 5, and 10 μg/μL of aurantiamide acetate were used for standard curve construction (Supplementary Figure S1A,B).

### 2.3 *In vitro* transdermal permeation and absorption detection

The transdermal permeation assay was performed using a Franz diffusion chamber, as previously reported (Migdadi et al., 2018). The volume of the receiving diffusion chamber was 17 mL, and the effective permeation area was 1.67 cm^2^. The stratum corneum of the skin was kept facing upward throughout the procedure. A total of 600 µL of hydrogel was evenly applied to the dissected rat skin, and 200 µL of the receiving solution was sampled from the sampling tube at predetermined time points. 24 h after administration, the stratum corneum was removed, and the epidermis and dermis were collected separately, sonicated, and centrifuged, as reported previously (Wang et al., 2010; Zhang et al., 2023). The supernatant was filtered, and the epidermal and dermal retention of aurantiamide acetate was determined via UHPLC-Q-TOF-MS/MS (Supplementary Figure S1C,D).

### 2.4 Animal model and pharmacological effectiveness of PO

Both male and female C57BL/6J mice, weighing 19–22 g and aged 6–8 weeks, were provided by BesTest Bio-Tech (Zhuhai, China). DNCB, or 2,4-dinitrochlorobenzene (CAS: 97–00–7), was purchased from Millipore Sigma (237329, MO, United States). The mice were randomly divided into 5 groups: the vehicle control group (Ctr), DNCB-induced AD-like model group (DNCB), positive control group (0.75 mg dexamethasone/mL cream, Sanjiu Medical & Pharmaceutical, H44024170, DXMS), PO low-dose group (PO aqueous extract 10 mg/mL in carbomer hydrogel, PO-L), and PO high-dose group (PO aqueous extract 20 mg/mL in carbomer hydrogel, PO-H). Except for the Ctr group, the remaining 4 groups of mice were subjected to DNCB challenge. The Ctr and DNCB groups were treated with 0.9% physiological saline only. In another set of study, we included the carbomer hydrogel alone to compare the effects of carbomer and PO-H treatment. All animal care and experimental procedures were approved by the Animal Ethical Committee of the Department of Health and Supervision and carried out following the Institutional Animal Care and User Committee Guidelines of the Macau University of Science and Technology (SKLSOP/AD003/V3/20180619).

**FIGURE 2:**
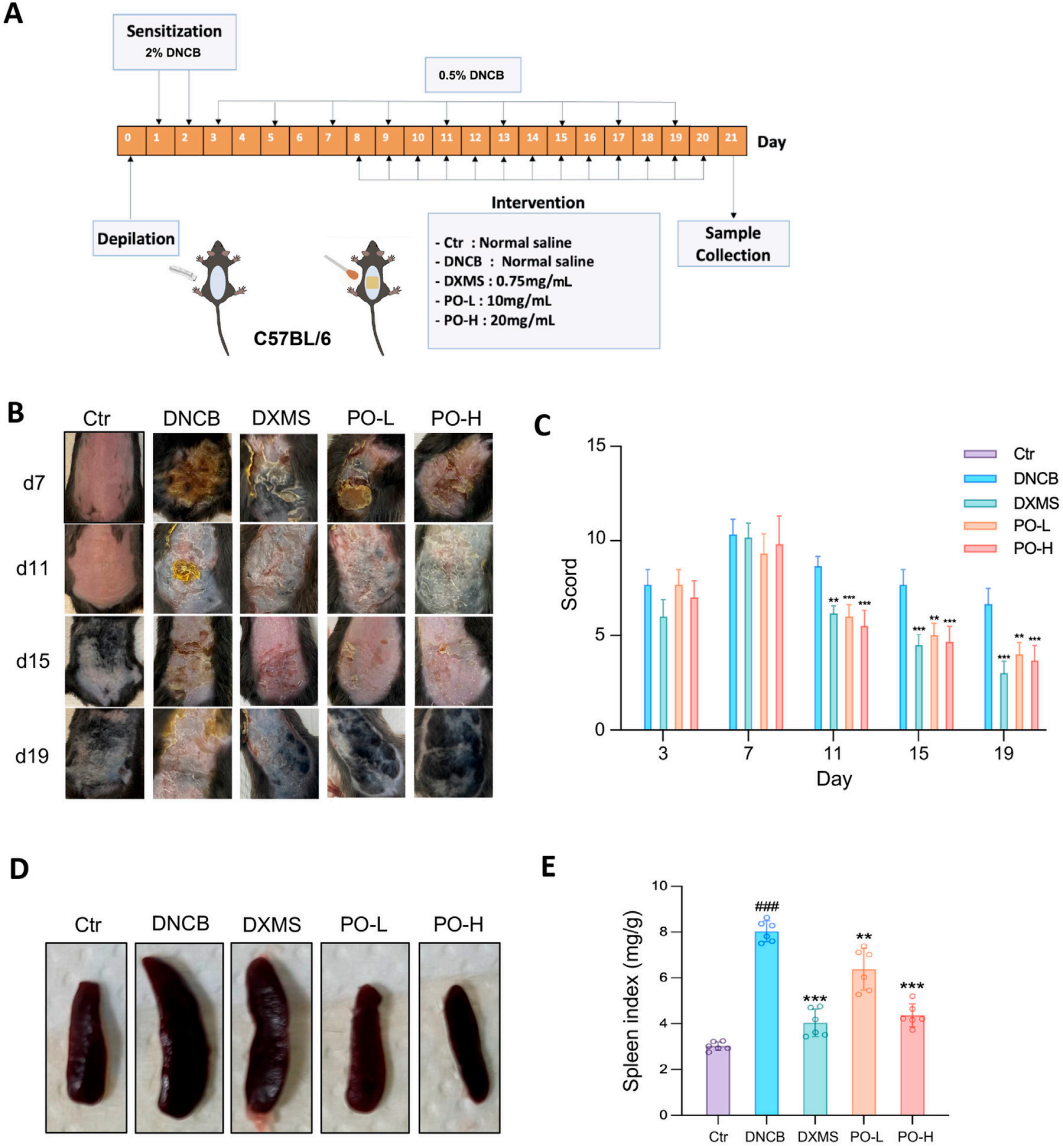
PO carbomer hydrogel attenuates DNCB-induced AD clinical symptoms in mice. **(A)** Schematic diagram of the animal study protocol. **(B)** Representative photographs of the dorsal skin lesions on the 7th, 11th, 15th, and 19th days of the experiment. **(C)** SCORAD scores were assessed every 4 days. **(D,E)** Representative photographs of the spleen and the spleen indices of the mice. The data are expressed as means ± SD, n = 6. ^###^ *p* < 0.001 vs. the vehicle control (Ctr) group; ^*^ *p* < 0.05, ^**^ *p* < 0.01, and ^***^ *p* < 0.001 vs. the DNCB-induced AD group.

The AD-like mouse model was established as previously described (Bai et al., 2023; Liu et al., 2023) with some modifications, and the schematic protocols are shown in Figure 2A. Briefly, 1 day before the study, the mouse’s back skin (2 × 1.5 cm^2^) was depilated with hair removal cream. DNCB was prepared in a mixture of acetone and olive oil (4:1, v:v). For the first 2 days of sensitization, 100 μL of 2% DNCB was evenly applied to the dorsal skin. From day 3 until day 21, sensitization was performed with 100 μL of 0.5% DNCB every other day. Equal volumes of acetone and olive oil mixture were used in the Ctr group. From days 8–21, the mice in the DXMS and two PO groups were topically treated (600 μL/day).

On days 7, 11, 15, and 19, images of the dorsal skin were captured via a previously described imaging protocol (Liu et al., 2023). SCORing of atopic dermatitis (SCORAD) was recorded on days 3, 7, 11, 15, and 19 as 0–3 according to severity on the basis of four skin symptoms (erythema/hemorrhage, edema, lichenification/dryness, and ulceration), as previously reported (Bai et al., 2023). 12 h after the last treatment on day 21, mice were euthanized by CO_2_. Each mouse was carefully observed for signs of unconsciousness, typically within 2–3 min. CO_2_ flow was maintained for at least 1 min after the cessation of respiration to ensure complete euthanasia. The spleen was then dissected, and the spleen index, the ratio of the spleen weight to the body weight (mg/g), was calculated. Blood was withdrawn, and the serum was prepared by centrifugation and stored at −80°C. Half of the dorsal skin in the sensitized area was collected for histologic and immunostaining studies. The other half of the skin was snap-frozen in liquid nitrogen and stored at −80°C.

### 2.5 Histological analysis and immunostaining

The skin tissues were processed routinely and embedded in paraffin for staining with hematoxylin and eosin (HE). Toluidine blue staining (Solarbio, G3670, Beijing, China) was used to identify mast cell infiltration, and Masson’s trichrome stain (Solarbio, G1340, Beijing, China) was used to determine the collagen fiber content in affected areas. The stained tissue was observed and measured in five random fields via a MoticEasyScan Pro 6 system (Motic Tokyo, Japan). The epidermal thickness was measured by ImageJ 1.48v (Rawak Software, Inc. Germany).

For immunohistochemical staining, paraffin-embedded tissue sections were deparaffinized, rehydrated, and subjected to antigen retrieval. After permeabilization, the sections were incubated overnight at 4°C with a rabbit polyclonal anti-CD4 antibody (#0647R, Bioss, Beijing, China) at a 1:300 dilution, followed by incubation with horseradish peroxidase-labeled goat anti-rabbit IgG and development with 3, 3′-diaminobenzine-chromogen substrates. One representative section from each mouse and five fields from each section were randomly selected for histological evaluation. ImageJ software was used to determine the positive area.

For immunofluorescence staining, the sections were incubated with an anti-filaggrin primary antibody (1:300, #6327R; Bioss, Beijing, China) overnight at 4°C. The next day, the sections were incubated with an Alexa Fluor 555-labeled anti-rabbit secondary antibody (Invitrogen) (1:300) at 37°C for 30 min. Subsequently, 4′,6-diamidino-2-phenylindole solution (DAPI, 62247, Thermo Scientific, Waltham, MA, United States) was added to stain the nuclei at 37°C for 15 min. Finally, the sections were sealed with buffered glycerol and visualized under a fluorescence microscope (CKX53, Olympus, Japan).

### 2.6 Cell culture and viability assay

HaCaT keratinocytes were cultured in Dulbecco’s modified Eagle medium (Gibco, Invitrogen, NY, United States) supplemented with 10% fetal bovine serum (Gibco, Invitrogen, NY, United States) and 1% penicillin/streptomycin (Gibco, Invitrogen, NY, United States) at 37°C in a 5% CO_2_ incubator. HaCaT cells were pretreated with aurantiamide acetate (25, 50, or 100 μM) or vehicle (0.1% DMSO) for 1 h before tumor necrosis factor-alpha (TNF-α, HY-P7058, MCE, United States) and interferon-γ (IFN-γ, HY-P7025, MCE, United States) challenge for 18 h. During the viability assay, the cells were exposed to various concentrations of aurantiamide acetate for 12 h. Cell viability was determined with a Cell Counting Kit-8 (MA0218, MeilunBio, Dalian, China).

### 2.7 Enzyme-linked immunosorbent assay

The skin tissue supernatant and serum were used for the measurement of IgE (Lot#JL12885), IL-4 (Lot#JL20266), IFN-γ (Lot#JL10967), and IL-17 (Lot#JL20250) levels via enzyme-linked immunosorbent assay (ELISA) kits (JiangLai, Shanghai, China) according to the manufacturer’s instructions.

### 2.8 RNA extraction and quantitative real-time PCR

Total cellular RNA was extracted via the Steadypure RNA Extraction Kit (Accurate Biotechnology, Hunan, China). After treatment with DNase I, RNA quantity was assessed via a Nanodrop One spectrophotometer (Thermo Scientific, Wilmington, DE, United States). cDNA was synthesized via an Evo M-MLV RT Kit (Accurate Biotechnology, Hunan, China). Quantitative real-time PCR was performed via a thermal cycler (CFX Opus 96, Bio-Rad, United States) with SYBR Green Pro master mix (Accurate Biotechnology, Hunan, China). Glyceraldehyde 3-phosphate dehydrogenase (GAPDH) and β-actin were used as internal controls. Melting curves were generated upon completion of the cycles to ensure the absence of nonspecific products. The data were analyzed via the 2^−△△CT^ method and are presented as the fold change in the transcript levels of the target genes normalized to those of GAPDH or β-actin. The primers used were acquired from BGI (BGI Genomics Co., Ltd., Beijing) and are listed in Supplementary Table S1.

### 2.9 Western blotting

Cell/tissue protein was extracted with RIPA buffer (ab156034, Abcam, Shanghai, China) in the presence of protease inhibitors (Roche Diagnostics, Indianapolis, IN, United States) and phosphatase inhibitors (Thermo Scientific, 32957, Waltham, MA, United States) and quantified with a bicinchoninic acid protein assay kit (Thermo Scientific, 23227, Waltham, MA, United States). Proteins were electrophoresed on 7%–10% sodium dodecyl sulfate‒polyacrylamide gels and transferred to nitrocellulose filter membranes. After blocking, the membrane was incubated with the following primary antibodies (1:1000) at 4°C overnight: JAK1 (#3344), p-JAK1 (#3331), STAT3 (#9139), p-STAT3 (#9145), STAT6 (#5397), β-actin (#8457), and GAPDH (#5174) from Cell Signaling Technology (MA, United States); p-STAT6 (ab263947) and IL-17 (ab79056) purchased from Abcam (Shanghai, China); and Ki-67 (#2130R), Caspase-14 (#10136R), filaggrin (#6327R), and CD4 (#0647R) obtained from Bioss (Beijing, China). The membrane was then incubated with the respective secondary antibodies: goat anti-rabbit (or anti-mouse) horseradish peroxidase-conjugated secondary antibodies (7074 and #7076; Cell Signaling Technology, MA, United States). The blots were developed in ECL-plus detection reagent (4AW012, 4A Biotech, Beijing) and visualized with an image analyzer (Amersham ImageQuant 800, Cytiva).

### 2.10 Network pharmacology analysis

Twenty ingredients in PO were retrieved from the TCMSP database (https://old.tcmsp-e.com/tcmsp.php) or the literature. Seven of these ingredients were validated by UHPLC-Q-TOF-MS/MS in PO aqueous extract (Supplementary Table S2). Swiss Target Prediction (https://www.swisstargetprediction.ch/) was used to predict the targets of 20 active ingredients. The disease targets were searched via “Atopic Dermatitis” as a keyword from the DisGeNET (https://www.disgenet.org/) and GeneCards (https://www.genecards.org/) databases. The data were standardized via UniProt (http://www.uniprot.org/). Venny 2.1.0 (https://bioinfogp.cnb.csic.es/tools/venny/) was used to obtain the intersection targets of the ingredients and diseases. STRING 12.0 (https://string-db.org/) was used for protein‒protein interaction (PPI) network profiling. DAVID (https://david.ncifcrf.gov/home.jsp) was utilized for enrichment analysis of the Kyoto Encyclopedia of Genes and Genomes (KEGG) pathway. An online bioinformatics platform (https://www.bioinformatics.com.cn) and Cytoscape 3.10.2 were used for analysis and visualization.

### 2.11 Molecular docking analysis

The chemical structures of the PO ingredients were downloaded from the PubChem database. The 3D structure of the JAK1 protein (6BBU) was downloaded from the RCSB Protein Data Bank. Autodock Tools 1.5.6 were used to analyze the rotatable bonds of the compounds and to remove ligands, water, and hydrogen atoms from the protein structures. AutoDock 4.2.6 and PyMOL 3.0.1 were used for molecular docking and visualization, respectively.

### 2.12 siRNA transfection

HaCaT cells were plated in 6-well plates the day before transfection and reached 70% confluence on the day of transfection. The cells were transfected with small interfering RNA (siRNA) via the siRNA-Mate Plus transfection reagent (G04026; GenePharma, Shanghai, China) according to the manufacturer’s instructions. siRNAs targeting human JAK1 (sense-5′-GGA CCC UGA UGG AUU ACA ATT-3′; antisense 5′- UUG UAA UCC AUC AGG GUC CTT-3′) or scrambled RNA (negative control (NC) siRNA) were applied at 1.5 μM in Opti-MEM reduced serum medium (31985, Invitrogen). The silencing efficiency was verified via quantitative real-time PCR. 24 h after siRNA transfection, the cells were pretreated with aurantiamide acetate (100 μM) for 1 h prior to TNF-α and IFN-γ challenge for 18 h.

### 2.13 Inhibition of JAK1 tyrosine kinase activity

A JAK1 Assay Kit (79518, BPS Bioscience, San Diego, United States) was used to measure the inhibitory effects of aurantiamide acetate on JAK1 tyrosine kinase activity. The assay employed ADP-Glo™ as a detection reagent (V6071, Promega) and was conducted via a luminescence plate reader (SpectraMax iD3, Molecular Devices) following the manufacturer’s instructions.

### 2.14 Total antioxidant capacity test *in vitro*

The total antioxidant capacity (T-AOC) was determined by the rapid ABTS (2,2′-azino-bis (3-ethylbenz-thiazoline-6-sulfonic acid) method using the T-AOC Assay Kit (S0121, Beyotime, Shanghai, China). The antioxidant capacity was evaluated by measuring the absorbance at a wavelength of 414 nm.

### 2.15 Detection of malondialdehyde (MDA) content

The content of MDA in skin homogenate was measured using the Lipid Peroxidation MDA Assay Kit (S0131S, Beyotime, Shanghai, China). All procedure was performed according to the manufacturer’s instruction. The MDA content per mg wet tissue was evaluated by measuring the absorption value at 532 nm.

### 2.16 Measurement of wet-to-dry weight ratio of the skin

The affected skin was collected using a 10 mm-diameter punch and then dried in an oven at 65°C for 72 h until a constant weight was achieved. The wet-to-dry weight ratio was then calculated.

### 2.17 Quantification and statistical analysis

The results are expressed as the means ± SD. Student’s t*-*test and one-way ANOVA with *post hoc* Dunnett’s multiple comparison were used for normally distributed variables. Nonparametric Kruskal‒Wallis tests were used for nonnormally distributed variables. And repeated data were compared using repeated measures analysis of variance. All the cell culture data are representative of 3 independent replicate experiments. For *in vivo* studies, the n value corresponds to the individual animal of a given treatment. Values of *p* < 0.05 were considered statistically significant. The statistical analysis was performed with GraphPad Prism V9 (GraphPad Software, La Jolla, CA, United States).

## 3 Results

### 3.1 Preparation, characterization and epidermal absorption of PO aqueous extracts

The overall yield of the aqueous extract was 12.6% ± 0.4% (n = 3), and the carbomer hydrogel was subsequently prepared (Figure 1B). A total of 23 ingredients were identified by UHPLC-Q-TOF-MS/MS in the PO aqueous extract (Supplementary Table S2). The retention time of aurantiamide acetate in the PO extract was 15.32 min, which was consistent with that of the reference compound (Figure 1C). The relative content of aurantiamide acetate in the PO aqueous extract is 6.1 ± 0.3 mg/mL (n = 3), which serves as a standardized parameter to ensure the quality of the PO aqueous extract. This high abundance, constituting 1.5% ± 0.1% (n = 3) of the fresh PO plant, prompted us to further explore its drug candidacy as an active ingredient.

We then demonstrated that the PO aqueous extract-carbomer hydrogel formulation provided stable drug absorption into the epidermis and dermis. The drug content, measured by aurantiamide acetate, in the receiving solution of the Franz diffusion chamber steadily increased over 24 h following the topical administration of the carbomer hydrogel to isolated skin tissue (Figure 1D). Further analysis revealed that the retention rates of aurantiamide acetate in the epidermis and dermis were 65.8 ± 14.7 and 19.7 ± 5.9 μg/cm^2^, respectively (Figure 1E). The epidermal and dermal retention rates were 11.8% and 3.5%, respectively, which are comparable to those of the current treatment (Phatale et al., 2022). Additionally, we tested the *in vitro* total antioxidant capacity of the PO hydrogel. The PO hydrogel containing 0.2 mg aqueous extract exhibited a Trolox-equivalent antioxidant capacity of 0.89 ± 0.12 mM (Figure 1F). Overall, we provide a simple method to prepare aqueous extracts and introduce a hydrogel formulation that can be readily applied for the treatment of skin diseases.

### 3.2 PO hydrogel improved DNCB-induced clinical symptoms in mice

An AD-like model was successfully established in mice by repeated DNCB sensitization (Figure 2A), where erythema, swelling, lichenification, crusting, and even severe ulcers were evident in dorsal skin challenged with DNCB (Figure 2B), which is consistent with previous reports (Bai et al., 2023; Liu et al., 2023; Kim et al., 2024). These dorsal skin symptoms were significantly alleviated by the PO hydrogel (Figure 2B). The SCORAD of the DNCB group markedly increased from day 11 until day 19, which was significantly attenuated by approximately 30%–40% with the topical application of the PO hydrogel. Moreover, these beneficial effects persisted until the day before sacrifice, given continuous DNCB sensitization (Figure 2C). Next, the spleen mass and index were measured to assess the overall immune response to the dorsal skin lesions and the effectiveness of the treatment. We found that DNCB-induced AD mice presented enlarged spleens and a significant increase in the spleen index (Figures 2D,E), with the latter being dramatically reduced in PO-L- and PO-H-treated mice by 14% (*p* < 0.01) and 59% (*p* < 0.001), respectively (Figure 2E).

We further compare the therapeutic efficacy of PO hydrogel versus carbomer alone in alleviating DNCB-induced AD clinical symptoms and cutaneous hydration. SCORAD scoring showed an approximate 40% reduction in erythema/edema in PO-H group (*p* < 0.001) versus 10% reduction in carbomer alone group (*p* = 0.064) (Supplementary Figure S2A,B). Carbomer hydrogel alone did not lower the spleen index (7.08 ± 2.09 *vs.* 5.69 ± 1.31 mg/g, *p* = 0.14), while PO-H treatment (3.69 ± 0.49 mg/g) was able to further significantly reduce the spleen index relative to carbomer alone (*p* = 0.002) (Supplementary Figure S2C). Compared with DNCB group (4.52 ± 0.32), both carbomer hydrogel (4.09 ± 0.10, *p* = 0.008) and the PO-H group (3.57 ± 0.18, *p* < 0.001) significantly reduced the wet/dry ratio of the dorsal skin. Moreover, the dorsal skin wet/dry ratio of the PO-H group was lower than that of the carbomer hydrogel group (*p* < 0.001) (Supplementary Figure S2D). These findings suggest that the therapeutic effects of PO are attributed not to mere moisturization but to the ability to counteract inflammatory exudation, especially during the acute stage of the induced severe AD.

### 3.3 PO hydrogel attenuated pathological skin impairments in mice

**FIGURE 3:**
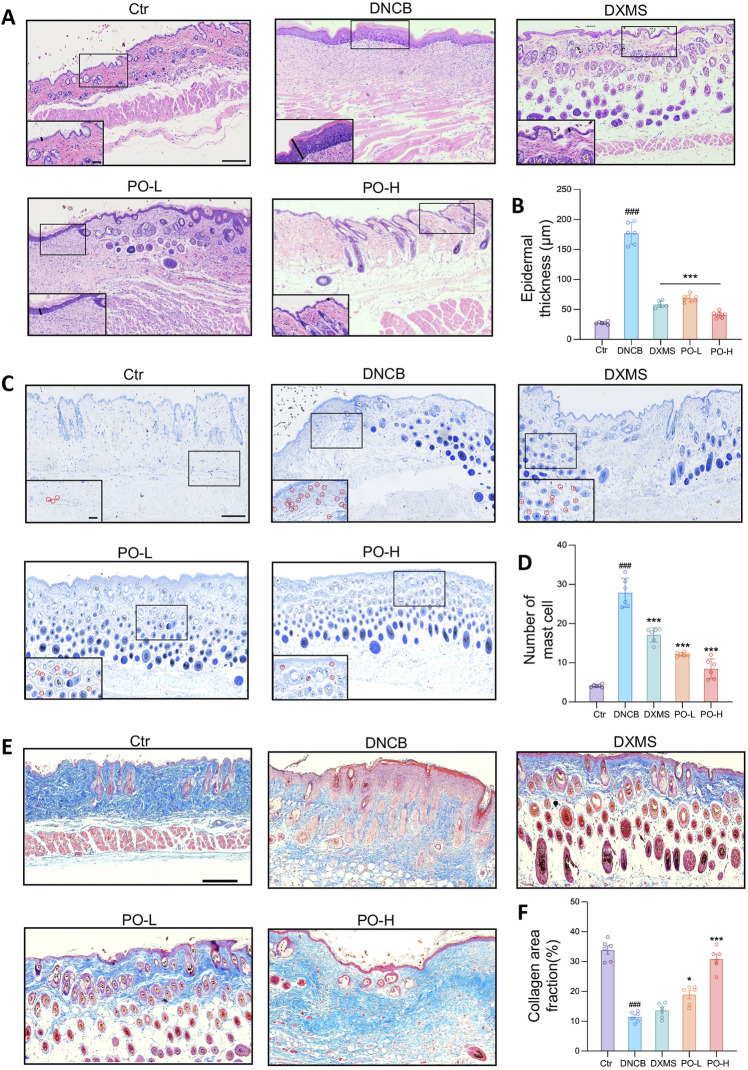
PO carbomer hydrogel mitigated histopathological defects in mice with DNCB-induced AD. **(A)** Representative HE-stained dorsal skin on the 21st day of the experiment. The epidermal thickness was determined as indicated by the solid black lines. **(B)** Measurement of epidermal thickness as indicated in **(A)**. **(C)** Representative toluidine blue-stained mast cells, as indicated by the red circles in the insets. **(D)** The number of mast cells per random microscopic field of the dorsal skin (×400 magnification). **(E)** Representative Masson’s trichrome-stained dorsal skin on the 21st day of the experiment. **(F)** Calculated collagen area fraction (blue). The data are expressed as means ± SD. ^###^ *p* < 0.001 vs. the vehicle control (Ctr) group; ^*^ *p* < 0.05, ^**^ *p* < 0.01, and ***p < 0.001 vs. the DNCB-induced AD group. Scale bars indicate 200 μm and 60 μm (insets of higher magnification), respectively.

The infiltration of lymphocytes and neutrophils was significantly increased in the dorsal skin of DNCB-induced AD mice. However, PO hydrogel intervention partially diminished the infiltration of inflammatory cells in the epidermis and dermis, as shown in HE-stained tissues (Figure 3A). Compared with the DNCB group, the PO hydrogel group decreased epidermal thickness by 1.7 and 3.9 folds in PO-L and PO-H groups, respectively (both *p* < 0.001) (Figure 3B). Mast cell proliferation was significantly greater in the DNCB group than in the Ctr group, whereas PO treatment dose-dependently reduced mast cell proliferation (Figure 3C). The number of mast cells in the DNCB group increased by 4.7-fold compared to the control group (*p* < 0.001). PO treatment dosedependently inhibited the proliferation of mast cells (PO-L by 1.0 and PO-H by 2.0 folds, both *p* < 0.001) (Figure 3D). The content of collagen, a key component of skin barrier integrity, was dramatically reduced 21 days after DNCB sensitization. The collagen content was significantly increased after PO treatment, as indicated both qualitatively by Masson’s trichrome stain (Figure 3E) and quantitatively by the collagen area fraction (Figure 3F). For example, PO-H treatment dramatically increased the collagen area fraction by 1.9 folds compared to the DNCB group (*p* < 0.001) (Figure 3F).

### 3.4 PO hydrogel alleviates cutaneous inflammation in AD mice

**FIGURE 4:**
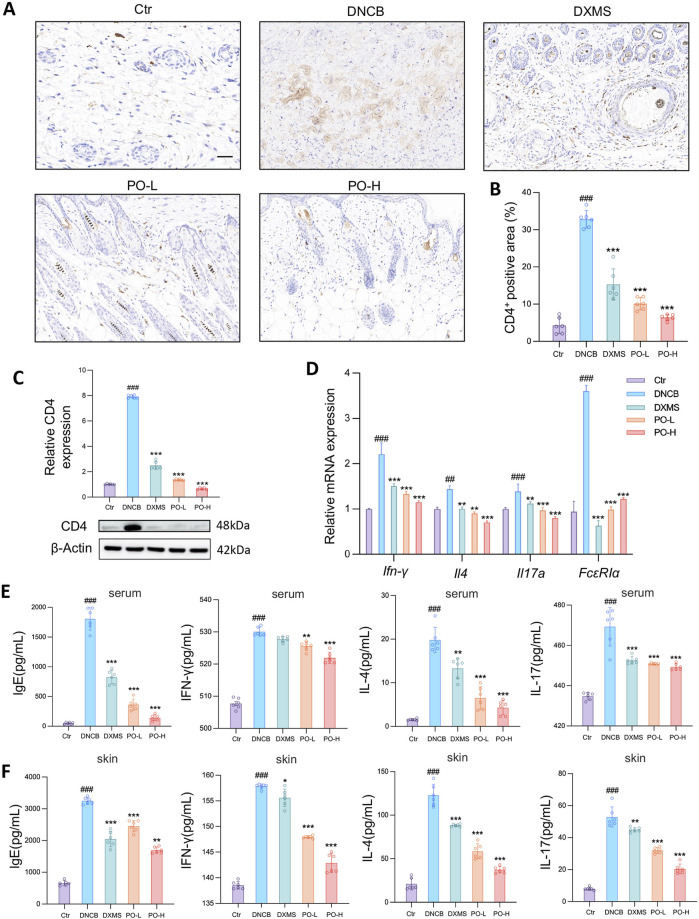
PO carbomer hydrogel modulates cutaneous immune responses and suppresses the generation of proinflammatory factors in DNCB-induced AD mice. **(A)** Immunohistochemical staining of CD4^+^ cell infiltration (brown) in the skin. The scale bar represents 100 μm. **(B)** Quantification of the CD4^+^ area in the skin. **(C)** Relative CD4 protein expression in dorsal skin. **(D)** Relative mRNA expression of *Ifn-γ*, *Il4*, *FcεRIα,* and *Il17a* in dorsal skin, as detected by quantitative PCR. n = 6. **(E,F)** Determination of IgE, INF-γ, IL-4, and IL-17 concentrations in mouse serum **(E)** and affected skin **(F)** via ELISA. The data are expressed as means ± SD. ^#^ *p* < 0.05, ^##^ *p* < 0.01, ^###^ *p* < 0.001 vs. the vehicle control (Ctr) group; ^*^ *p* < 0.05, ^**^ *p* < 0.01, and ^***^ *p* < 0.001 vs. the DNCB-induced AD group.

Immunohistochemical staining revealed that CD4^+^ T-cell infiltration was significantly increased by 7 folds in the DNCB group (Figure 4A), whereas PO-L and PO-H treatment significantly reduced the CD4^+^ T-cell infiltration area by 1.8 and 2.9 folds relative to DNCB, respectively (Figure 4B) and protein expression of CD4 by 3.7 and 7.9 folds (Figure 4C). Additionally, the mRNA levels of *Ifn-γ*, *Il4,* and *Il17a* were significantly increased by DNCB. However, these cytokine mRNA levels were significantly suppressed by approximately 50%–80% after PO treatment in a dose-dependent manner (Figure 4D). Notably, the DNCB group showed a 3.5-fold increase in FcεRIα mRNA expression compared to the Ctr group (*p* < 0.001). PO-H treatment demonstrated 1.9-fold inhibition of mast cell activation by hampering the expression of FcεRIα mRNA in the affected skin tissues (Figure 4D). Moreover, the production of IgE, IFN-γ, IL-4, and IL-17, both in the serum (Figure 4E) and in the dorsal skin (Figure 4F), was significantly reduced by PO hydrogel treatment.

### 3.5 PO hydrogel restores the skin barrier in AD mice

**FIGURE 5:**
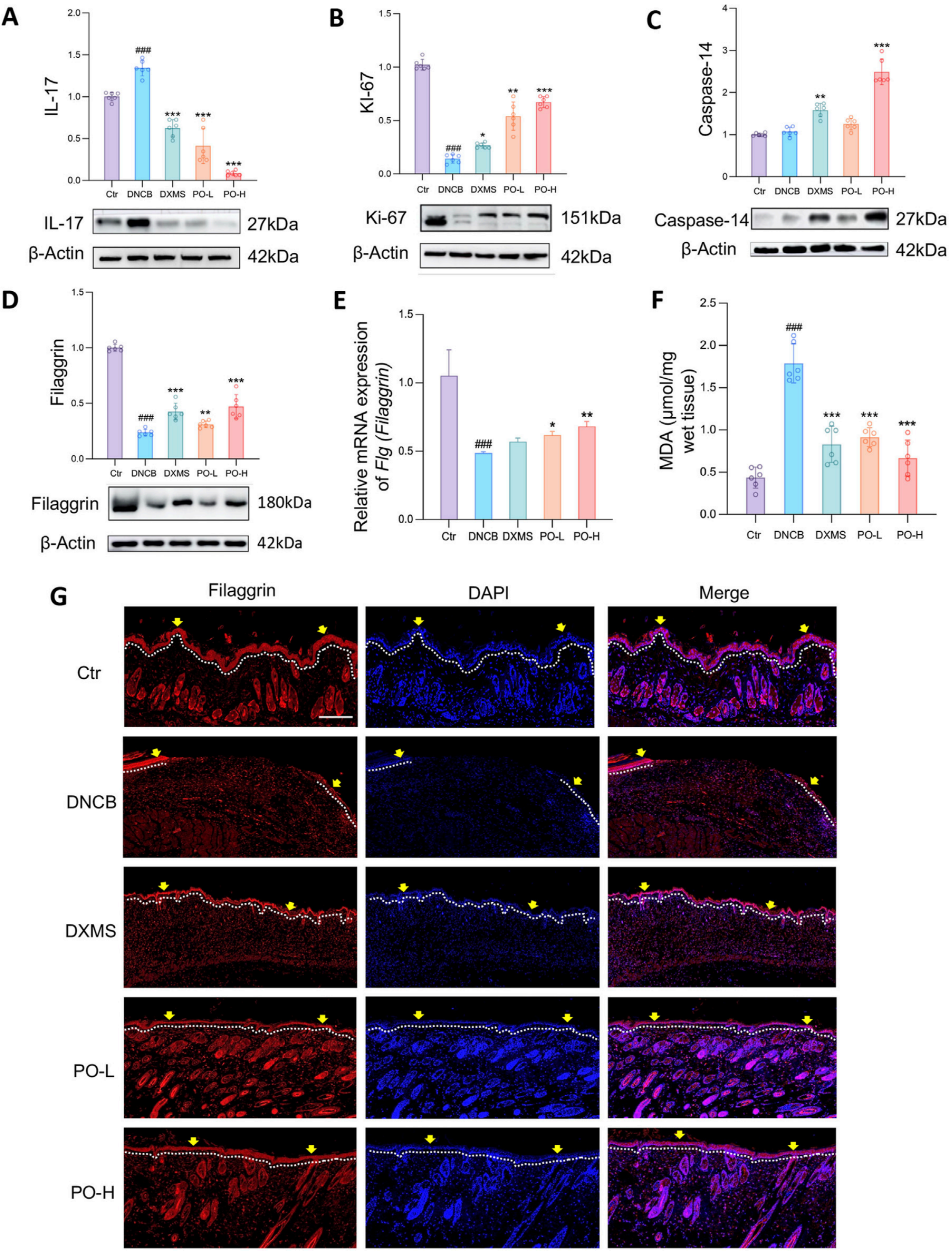
PO carbomer hydrogel modulates immune responses and partially restores the damaged skin barrier. **(A–D)** Relative protein expression of IL-17, Ki-67, caspase-14, and filaggrin was detected by Western blotting. Blots are representative of 6 independent experiments. **(E)** Relative mRNA expression of *Flg (Fillagrin)* detected by quantitative PCR in the dorsal skin. n = 6. **(F)** Content of MDA in the affected skin. n = 6. MDA, malondialdehyde. **(G)** Immunostaining of dorsal sections for filaggrin (red) and nuclei (blue). The epidermis is shown along the white dotted lines. The yellow arrows indicate the filaggrin-derived skin barrier in the epidermis. Scale bar = 100 μm. The data are expressed as means ± SD. ^#^ *p* < 0.05, ^##^ *p* < 0.01, ^###^ *p* < 0.001 vs. the vehicle control (Ctr) group; ^*^ *p* < 0.05, ^**^ *p* < 0.01, and ^***^ *p* < 0.001 vs. the DNCB-induced AD group.

IL-17 protein expression was significantly elevated by in the skin of DNCB-challenged mice (Figure 5A), primarily due to local Th17 accumulation and activation (Bakker et al., 2023). IL-17 protein expression was suppressed by PO-L and PO-H hydrogel administration by 63% and 88%, respectively (both *p* < 0.001) (Figure 5A), which was consistent with previous transcriptional results. Additionally, Ki-67 and Caspase-14 protein levels were greater in the PO-H group than in the DNCB group by 64% and 97%, respectively (Figures 5B,C). Additionally, Ki-67 acts as a marker for cell proliferation and tissue repair, while Caspase-14 increases the expression of filaggrin (Denecker et al., 2008), which subsequently contributes to the formation of the epidermal barrier (Irvine et al., 2011). Indeed, the PO hydrogel moderately restored the expression of filaggrin at both the translational (Figure 5D) and transcriptional stages in mouse dorsal skin (Figure 5E). Moreover, regional tissue peroxidation determined by MDA levels showed that DNCB-challenged skin showed a 4.1-fold increase compared to Ctr group (*p* < 0.001). PO treatment reduced MDA by 65% (PO-L) and 88% (PO-H) (*p* < 0.001 *vs.* DNCB), respectively (Figure 5F). Furthermore, the immunofluorescence results confirmed that filaggrin levels were significantly lower in the DNCB group than in the control group and that PO treatment partially restored filaggrin expression (Figure 5G). These findings provide evidence that the PO hydrogel improves epidermal integrity and helps restore skin barrier function in AD-like animals.

### 3.6 Aurantiamide acetate suppresses cutaneous inflammation and restores skin barrier function through targeted inhibition of JAK1

**FIGURE 6:**
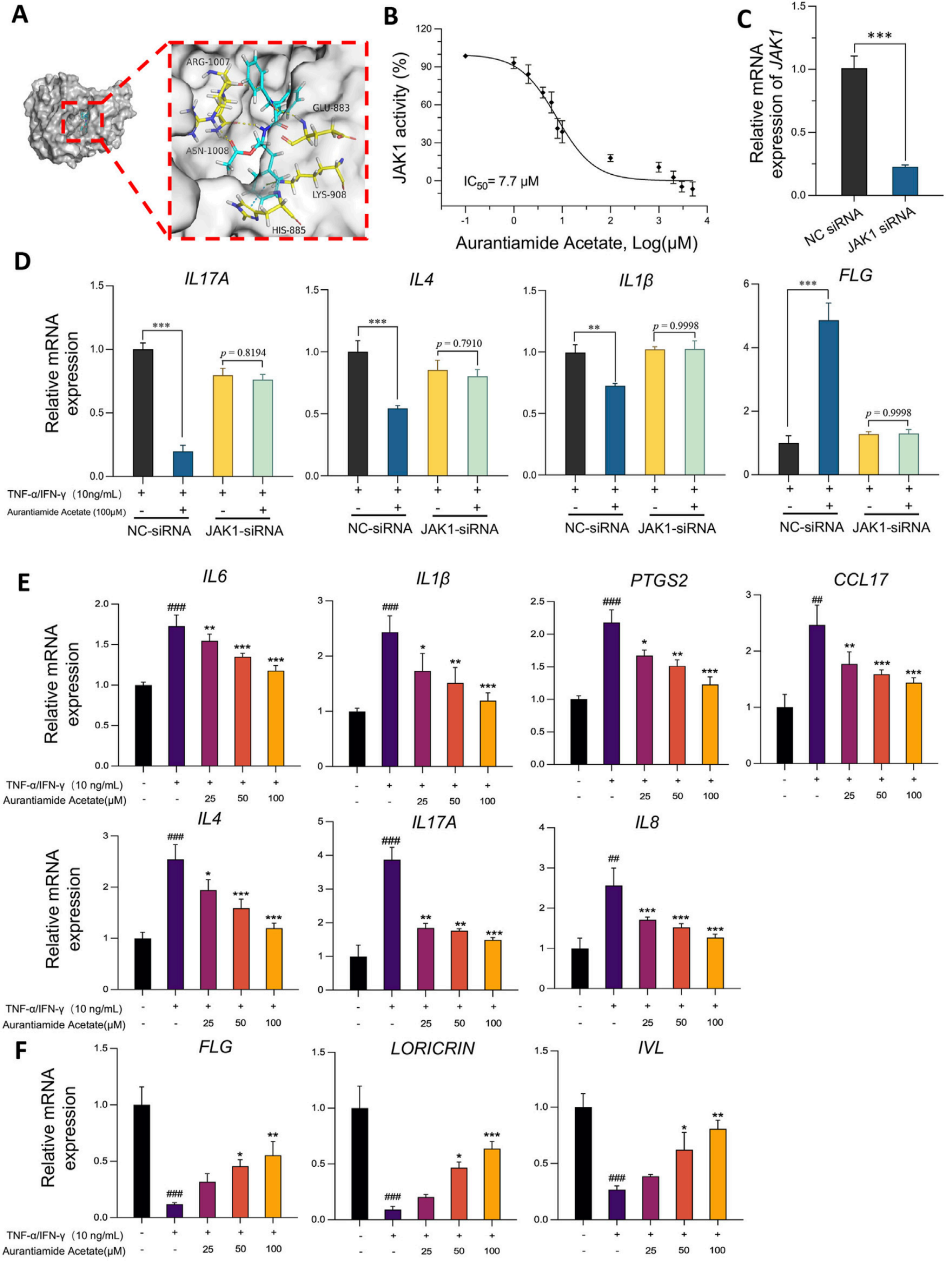
Aurantiamide acetate suppresses dermal inflammation and restores skin barrier function through targeting JAK1. **(A)** Diagram of the molecular docking between aurantiamide acetate, an active ingredient in PO, and JAK1 (PDB: 6BBU). **(B)** Inhibitory effects of aurantiamide acetate on JAK1 tyrosine kinase activity detected by the ADP-Glo™ reagent. **(C)** siRNA-JAK1 silencing efficiency 24 h after transfection. **(D)** Relative mRNA expression levels of *IL17A, IL4, IL1β* and *FLG* were detected via quantitative PCR in HaCaT cells transfected with NC or JAK1 siRNA. **(E)** Aurantiamide acetate inhibits proinflammatory gene transcription in non-siRNA-treated HaCaT keratinocytes stimulated with TNF-α/IFN-γ. **(F)** Aurantiamide acetate upregulates skin barrier protective gene transcription in non-siRNA-targeted HaCaT keratinocytes stimulated with TNF-α/IFN-γ. **(C–F)** Cells were pretreated with aurantiamide acetate (25, 50, or 100 μM) or vehicle (0.1% DMSO) for 1 h before TNF-α (10 ng/mL) and IFN-γ (10 ng/mL) challenge for 18 h. Relative mRNA expression levels of *IL6*, *IL1β*, *PTGS2 (COX-2)*, *CCL17 (TARC)*, *IL8*, *IL4*, *IL17A*, *FLG*, *LORICRIN* and *IVL* were detected via quantitative PCR. The data were normalized to internal GAPDH or β-Actin gene expression. The data are expressed as means ± SD of 3 independent experiments performed in duplicate. ^#^ *p* < 0.05, ^##^ *p* < 0.01, ^###^ *p* < 0.001 vs. the nonstimulated group; ^*^ *p* < 0.05, ^**^ *p* < 0.01, and ^***^ *p* < 0.001 vs. the vehicle group.

To identify potential targets of PO for AD treatment, a total of 2,185 AD-related targets were obtained from available databases, and 308 targets were also identified for PO-derived active ingredients. The resulting 103 common targets can be considered potential targets of PO for AD treatment (Supplementary Figure S3A). The JAK-STAT signaling pathway was one of the top 20 KEGG-enriched pathways among the 143 identified pathways (Supplementary Figure S3B). Specifically, JAK1 and STAT3 are possible targets, as revealed by network analysis (Supplementary Figure S3C) and protein‒protein interactions (Supplementary Figure S3D). Using available JAK1 inhibitors as references, one of the PO active ingredients, aurantiamide acetate, was predicted to be a potential JAK1 inhibitor, forming hydrogen bonds with the Asn1008, Arg1007 and Glu883 residues (Figure 6A). The docking energy of JAK1 with aurantiamide acetate was −7.71 kcal/mol, which was moderate among 11 JAK1 inhibitors (Supplementary Table S3). The inhibitory effects of aurantiamide acetate on JAK1 activity were further validated by detecting tyrosine kinase activity, with an IC_50_ of 7.7 µM (Figure 6B). These findings suggest that JAK1 is a possible target and that aurantiamide acetate could be a tentative JAK1 inhibitor, as supported by network pharmacological predictions, molecular docking studies, and enzymatic activity assays.

To determine the role of JAK1 in the attenuation of TNF-α- and IFN-γ-mediated cutaneous inflammation and skin barrier damage, we administered JAK1 siRNA to effectively knock down JAK1 mRNA expression, as confirmed by real-time quantitative PCR (Figure 6C). In cells transiently transfected with NC siRNA, aurantiamide acetate pretreatment still attenuated the expression of *IL17A, IL4,* and *IL1β* in response to TNF-α and IFN-γ stimulation (all *p* < 0.001). However, in cells transfected with JAK1 siRNA, no significant change in the mRNA expression of these genes was observed between cells pretreated with or without aurantiamide acetate (all *p* > 0.5, Figure 6D). Consistent with these results, *FLG* mRNA expression was significantly increased by 3.8-fold in NC siRNA-transfected cells treated with aurantiamide acetate but remained unaffected in JAK1-knockdown cells (Figure 6D). In summary, JAK1 knockdown abrogates the effects of aurantiamide acetate on dermal inflammation and skin barrier restoration.

We then studied the inhibitory effects of aurantiamide acetate on inflammation in non-siRNA-transfected keratinocytes *in vitro*. Aurantiamide acetate showed no evident cytotoxicity in HaCaT cells at concentrations up to 800 μM (Supplementary Figure S3E). Aurantiamide acetate significantly inhibited the mRNA expression of IL-6, IL-1β, prostaglandin-endoperoxide synthase 2 (PTGS2 or COX-2), C-C motif chemokine 17 (CCL17, TARC), IL-8, and IL-4 in TNF-α/IFN-γ-stimulated HaCaT cells in a concentration-dependent manner (Figure 6E). Moreover, aurantiamide acetate restored skin barrier function by dramatically increasing the transcript expression of the filaggrin, involucrin, and loricrin genes in TNF-α/IFN-γ-challenged HaCaT cells (Figure 6F).

### 3.7 PO hydrogel and aurantiamide acetate suppress JAK1-STAT3/6 signaling *in vivo* and *in vitro*

**FIGURE 7:**
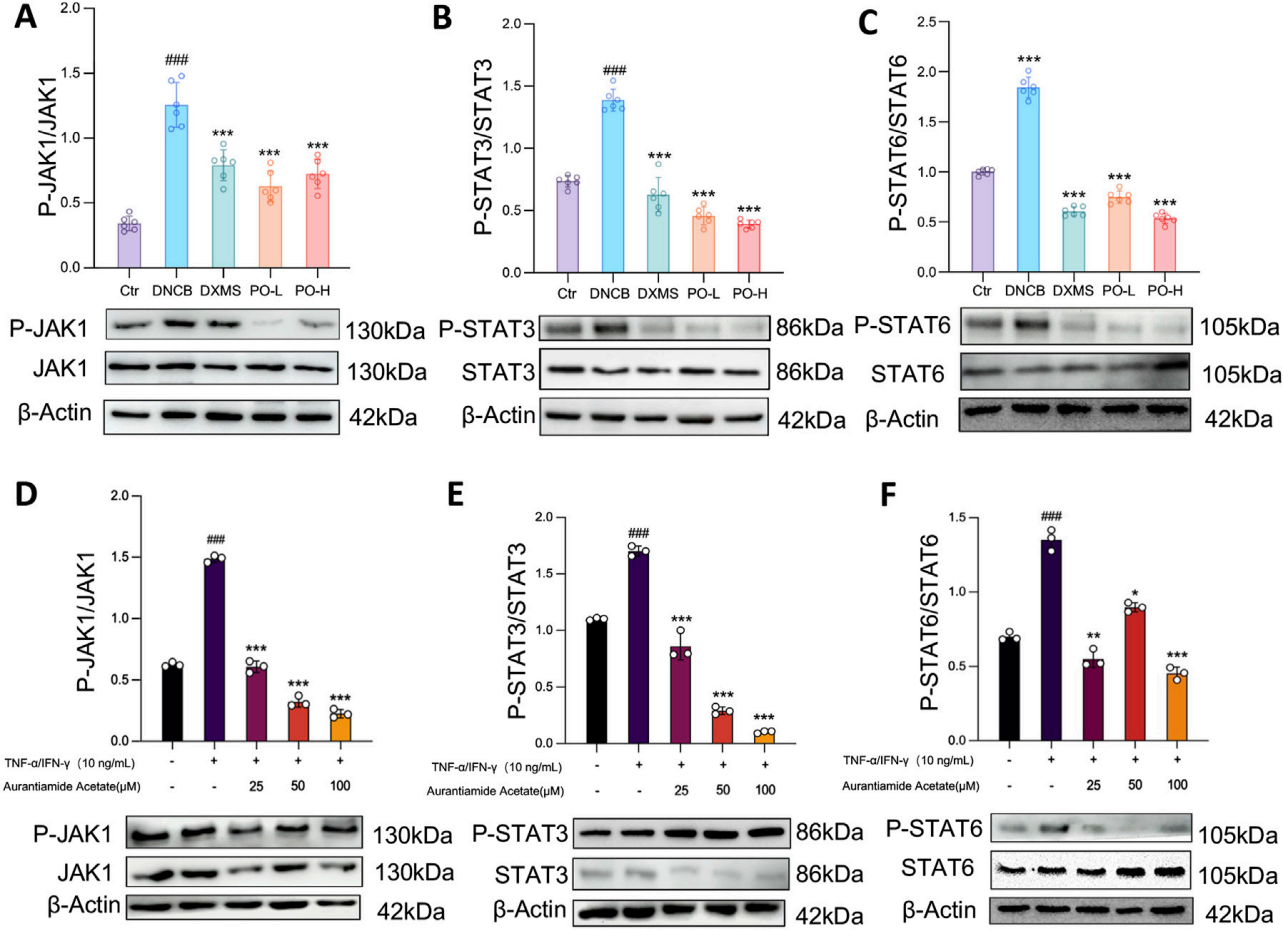
Aurantiamide acetate and PO carbomer hydrogel suppress the JAK1-STAT3/6 signaling pathway. **(A–C)** Western blot analysis of JAK1-STAT3/6 phosphorylation in mouse skin. n = 6. **(D–F)** Western blot analysis of JAK1-STAT3/6 phosphorylation in keratinocytes. The cells were pretreated with aurantiamide acetate (25, 50, or 100 μM) or vehicle (0.1% DMSO) for 1 h before being challenged with TNF-α (10 ng/mL) or IFN-γ (10 ng/mL) for 18 h. The data are expressed as means ± SD of 3 independent experiments performed in duplicate. ^#^ *p* < 0.05, ^##^ *p* < 0.01, ^###^ *p* < 0.001 vs. the Ctr/non-stimulated group; ^*^ *p* < 0.05, ^**^ *p* < 0.01, and ^***^ *p* < 0.001 vs. the vehicle/untreated group. The data were normalized to that of β-Actin.

Since activation of the JAK-STAT signaling pathway is correlated with the infiltration of inflammatory cells to accelerate AD progression (Gandhi et al., 2016; Chovatiya and Paller, 2021), we examined the effects of PO hydrogel or active compounds on signaling pathways by detecting posttranslational phosphorylation. The phosphorylation of JAK1 and STAT3/6 was significantly promoted in the DNCB group but was inhibited by PO hydrogel application (all *p* < 0.001) (Figures 7A–C). The *in vitro* results revealed that JAK1-STAT3/6 phosphorylation was also inhibited in cells pretreated with 25, 50, or 100 μM aurantiamide acetate (all *p* < 0.001) (Figures 7D–F).

## 4 Discussion

In this study, we simplified the preparation of PO aqueous extracts and introduced a hydrogel formulation, which demonstrated potent efficacy in attenuating DNCB-induced AD-like skin disease in mice. The PO hydrogel significantly ameliorated the severity of AD symptoms, suppressed cutaneous inflammation, and restored epidermal barrier function in animals. Mechanistically, we elucidated that PO and its active compounds improve epidermal barrier function and reduce inflammatory responses by inhibiting JAK1 activity and downstream STAT3/6 signaling pathways both *in vivo* and *in vitro*.

PO, known for its dual role as a medicinal and edible plant, has recently been reported to treat AD in animal models (Lv et al., 2022; Zhao et al., 2023). Traditionally, both fresh and dried plants have been used to prepare decoctions for topical and systematic administration. Our study introduced a simple extraction process to obtain a quality-controlled aqueous extract with stable permeation and absorption. The preparation method is more convenient and cost-effective than previous techniques, such as polysaccharide extraction (Lv et al., 2022) or fermentation (Zhao et al., 2023).

Epidermal barrier integrity and cohesion are crucial for maintaining skin hydration and protecting against microbes, irritants, and allergens. Defects in the epidermal barrier were observed in our DNCB-sensitized mice, which is consistent with our expectations and previous studies (Liu et al., 2023; Kim et al., 2024). Two weeks of topical application of the PO carbomer hydrogel maintained epidermal integrity by restoring the expression of crucial structural proteins such as filaggrin, involucrin, and loricrin in the epidermis. These proteins play essential roles in facilitating epidermal cell differentiation and proper skin barrier formation (Strasser et al., 2015). The regulatory mechanisms of PO treatment involve the upregulation of Caspase14, a crucial modulator of skin barrier formation (Eckhart and Tschachler, 2011; Kubica et al., 2014). Additionally, the increased expression of Ki-67, a marker of cellular proliferation, in PO-treated dorsal skin and pretreated keratinocytes suggests that the damaged tissues in AD mice undergo mitotic processes to accelerate recovery from AD. During the progression of AD, we observed peroxidative reactions in the impaired dorsal skin tissue, and PO hydrogel has good antioxidant properties both *in vitro* and *in vivo*. Limiting the generation of reactive oxygen species in tissues and reversing tissue peroxidation has a good auxiliary effect on the treatment of AD (Jia et al., 2023; Wu et al., 2023).

In the DNCB-induced AD mouse model, we observed a significant increase in the number of CD4^+^ cells in the dorsal skin, accompanied by an overproduction of Th2/Th17 cytokines. Aberrant immune responses to Th1/Th2 and Th17 cells are thought to be key factors in the development of AD (Stander, 2021). Th2-mediated cytokines such as IL-4, IL-5, and IL-13 promote the production of allergen-specific IgE by B cells (Dekkers et al., 2023) through the JAK/STAT pathway (Furue et al., 2019). Moreover, mast cells produce numerous inflammatory molecules, including histamine, leukotriene, and interleukin, which can lead to pruritus and lichenoid lesions in AD (Wang et al., 2021). IL-17A, which is primarily generated via Th17 activation, is elevated in the peripheral blood of AD patients and AD lesions (Spidale et al., 2020). Furthermore, IL-17A can influence the expression of keratinocyte adhesion molecules by downregulating filaggrin, resulting in epidermal barrier dysfunction (Bonefeld et al., 2016). Future work requires the use of flow cytometry to specifically classify Th1/Th2 and Th17 cells during the development of AD, thus providing precise insights into the target of PO on AD.

In this study, the PO hydrogel not only significantly reduced IgE levels but also inhibited the proliferation and infiltration of mast cells, thereby mitigating pruritus and lichenoid lesions in AD model mice and rats. Moreover, the application of the PO hydrogel resulted in a significant decrease in the phosphorylation of JAK1 and downstream STAT3/6 activation. Further pharmacological exploration of TNF-α/IFN-γ-challenged keratinocytes demonstrated that aurantiamide acetate effectively restored skin barrier function, inhibited the inflammatory response, and regulated the cutaneous immune system through the JAK-STAT pathway. To further investigate the therapeutic effects of PO on AD, we employed UHPLC-Q-TOF-MS/MS to identify aurantiamide acetate as one of the active substances in PO. Previous studies have demonstrated that aurantiamide acetate has anti-inflammatory activity and immunomodulatory properties (Liu et al., 2015), which is consistent with our findings. We further revealed that aurantiamide acetate acts as a JAK1 kinase inhibitor with specific binding profiles. Moreover, we present solid evidence indicating that aurantiamide acetate potentially diminishes JAK1 kinase activity and phosphorylation. These findings provide a rationale for the further development of aurantiamide acetate as a potential candidate anti-AD drug, which may circumvent the adverse effects of the current clinical regimen. However, the inhibitory potency and specificity of aurantiamide acetate for JAK1 require further investigation.

## 5 Conclusion

Conventional therapeutic approaches have been effective in reducing chronic inflammation but may be insufficient for patients with severe AD. This study proposes a therapeutic strategy to restore epidermal barrier function by modulating inflammatory responses, mitigating peroxidative reaction, inhibiting JAK1 kinase activity, and targeting downstream STAT3/6 signaling pathways. PO, a traditional herbal medicine, possesses clinical potential to treat AD or AD-like symptoms.

## Data Availability

The original contributions presented in the study are included in the article/Supplementary Material, further inquiries can be directed to the corresponding authors.
