# Supplementary material for: Aqueous extracts of Portulaca oleracea L. alleviate atopic dermatitis by restoring skin barrier function
Source: Front Pharmacol. 2025 Jun 5;16:1591394. doi: 10.3389/fphar.2025.1591394 (PMC12176770; doi:10.3389/fphar.2025.1591394)
Supplement: Supplementary file 1 [file DataSheet1.pdf]

Aqueous extracts of *Portulaca oleracea L.* alleviate atopic dermatitis by restoring skin barrier function

Haobin Wei [a], Zhang Chen [a, b], Wenjie Lai [a, b], Wenxian Wang [a], Xiqing Bian [a, b], Limin Zhang [c, *], Xinzhi Li [a, b, *]

[a] School of Pharmacy, Faculty of Medicine & Laboratory of Drug Discovery from Natural Resources and Industrialization, [b] State Key Laboratory of Quality Research in Chinese Medicines, Macau University of Science and Technology, Taipa, Macao, China

[c] State Key Laboratory of Magnetic Resonance and Imaging, Innovation Academy of Precision Measurement Science and Technology, The Chinese Academy of Sciences, Wuhan, China

Supplementary materials

Supplementary Tables S1-3.

Supplementary Figures S1-3.

Table S1. Primers used in real-time PCRs

Gene (Mus musculus)	Primer	Sequence 5′-3′
Casp14 (Caspase-14)	F	ACGCATGTTCCGTTAGAA
	R	CACCATGTGCCATGAGTACCA
Flg (Filaggrin)	F	ATGTCCGCTCTCCTGGAAAG
	R	TGGATTCTTCAAGACTGCCTGTA
Gapdh	F	CATGGCCTTCCGTGTTCCTA
	R	ATGCCTGCTTCACCACCTTCT
Il4	F	GGTCTCAACCCCCAGCTAGT
	R	GCCGATGATCTCTCTCAAGTGAT
Ifn-γ	F	ATGAACGCTACACACTGCATC
	R	CCATCCTTTTGCCAGTTCCTC
Il17a	F	TTTAACTCCCTTGGCGCAAAA
	R	CTTTCCCTCCGCATTGACAC
FcεRIα	F	ATTGTGAGTGCCACCGTTCA
	R	GCAGCCAATCTTGCGTTACA
Gene (Homo sapiens)	Primer	Sequence 5′-3′
FLG (FILAGGRIN)	F	TCTGAAGAACCCAGATGATCCA
	R	CATCAAAAGAAACTCAGTAAAGTCCAA
LORICRIN	F	GAGTTGGAGGTGTTTTCCAGGG
	R	GCAGAACTAGATGCAGCCGGA
IVL (INVOLUCRIN)	F	CAGCAGTCATGTGCTTTTCCT
	R	TCCTCCAGTCAATACCCATCAG
CCL17 (TARC)	F	CACGCAGCTCGAGGGACCAATGTG
	R	TCAAGACCTCTCAAGGCTTTGCAGG
IL8	F	TTTTGCCAAGGAGTGCTAAAGA
	R	AACCCTCTGCACCCAGTTTTC
IL6	F	ACTCACCTCTTCAGAACGAATTG
	R	CCATCTTTGGAAGGTTCAGGTTG
PTGS2 (COX-2)	F	CTGGCGCTCAGCCATACAG
	R	CGCACTTATACTGGTCAAATCCC
IL1β	F	CATTGCTCAAGTGTCTGAAGC
	R	CATGGCCACAACAACTGACG
IL4	F	CGTGGTCAGTGCGGATAACTA
	R	TGGTGTGAACTGTCAGGTTTC
GAPDH	F	ACAACTTTGGTATCGTGGAAGG
	R	GCCATCACGCCACAGTTTC
β-ACTIN	F	ACAGAGCCTCGCCTTTGC
	R	ACATGATCTGGGTCATCTTCTCG

Table S2. List of 23 active ingredients in aqueous extract of *Portulaca oleracea L.* validated by UHPLC-Q-TOF-MS/MS

No.	***Phytoconstituents***	***Rt/min***	***Measured Values （m/z）***	***Ppm***	***Monitoring Mode***	***Molecular Formula***	***Structure***
1	*Dopamine	1.17	154.0863	0.69	$[M + H]^+$	$C_8H_{11}NO_2$	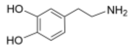
2	* (S)- (−)-salsolinol	1.20	180.102	0.72	$[M + H]^+$	$C_{10}H_{13}NO_2$	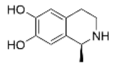
3	L-phenylalanine	2.06	166.0863	0.62	$[M + H]^+$	$C_9H_{11}NO_2$	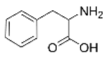
4	1-(furan-2-yl)-6,7-dihydroxy-3,4-dihydroisoquinoline	2.18	230.0818	0.164	$[M + H]^+$	$C_{13}H_{11}NO_3$	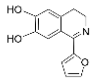
5	Oleraciamide A	2.47	329.1822	-4.48	$[M + H]^+$	$C_{17}H_{26}N_2O_3$	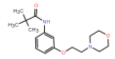
6	Portulacatone	2.56	230.0812	0.86	$[M + H]^+$	$C_{13}H_{11}NO_3$	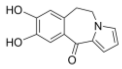
7	Indole-3-aldehyde	3.15	146.0602	0.72	$[M + H]^+$	C_9H_7NO	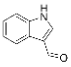
8	oleracone G	3.70	300.1230	7.02	$[M + H]^+$	$C_{17}H_{13}O_5^+$	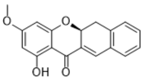
9	Oleracein E	3.85	220.097	0.84	$[M + Na]^+$	$C_{12}H_{13}NO_3$	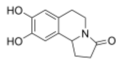
10	Rutin	4.20	611.1601	-0.29	$[M + H]^+$	$C_{27}H_{30}O_{16}$	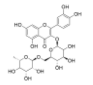
11	β-carboline	4.47	169.0761	-0.11	$[M + H]^+$	$C_{11}H_8N_2$	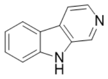
12	Oleracone B	4.58	207.1389	-0.75	$[M + H]^+$	$C_{13}H_{19}O_2$	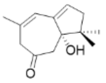
13	Oleracein D	5.21	696.2140	1.45	$[M + H]^+$	$C_{31}H_{38}O_{17}N$	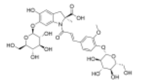
14	*Quercetin	5.89	303.0502	0.46	$[M + H]^+$	$C_{15}H_{10}O_7$	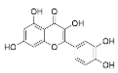
15	Oleracein K	6.25	844.2544	2.17	$[M + H]^+$	$C_{39}H_{40}NO_{19}$	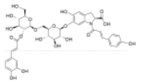
16	*Oleraciamide D	6.35	312.1234	1.07	$[M + H]^+$	$C_{18}H_{17}NO_4$	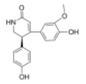
17	*Kaempferol	6.78	287.0548	-0.65	$[M + H]^+$	$C_{15}H_{10}O_6$	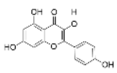

18	(R)-(+)-1-isobutyl-6,7-dihydroxy-1,2,3,4-tetrahydroisoquinoline	6.83	245.1384	-0.93	$[M + H]^+$	$C_{13}H_{20}NO_2$	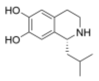
19	Ferulic acid methyl ester	6.97	209.0811	0.96	$[M + H]^+$	$C_{11}H_{12}O_4$	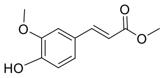
20	N-trans-feruloyloctopamine	7.76	330.1339	1.02	$[M + H]^+$	$C_{18}H_{19}NO_5$	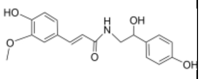
21	N-cis-feruloyltyramine	8.19	314.139	1.06	$[M + H]^+$	$C_{18}H_{19}NO_4$	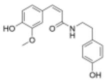
22	*Aurantiamide acetate	15.32	445.2125	0.86	$[M + H]^+$	$C_{27}H_{28}N_2O_4$	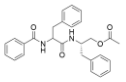
23	*Glycerol tributanoate	18.21	320.2069	0.64	$[M + H]^+$	$C_{15}H_{26}O_6$	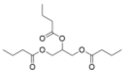

Note: *Selection of 7 compounds in network pharmacology analysis. Rt, retention time; Ppm, parts per million. These compounds are numbered in order based on retention time as shown in the chromatogram in Figure 1C.

Table S3. **Molecular docking between JAK1 inhibitors/Aurantiamide Acetate and JAK1 (PDB:6BBU)**

Code	Agonist	Docking Energy (Kcal/mol)	Salt Bridge Interaction	H-Bond Interaction	Pi-pi	Pi-cation
A1	Abrocitinib	-9.86	/	Glu957, Leu959, Glu966, Asn1008	/	/
A2	Upadacitinib	-9.29	/	Glu957, Leu959, Glu966, Asp1021	/	/
A3	SHR0302	-9.06	/	Glu957, Leu959, Glu966, Asp1021, Lys908	His885	/
A4	Ruxolitinib	-8.70	/	Glu957, Leu959	/	/
A5	Delgocitinib	-8.01	/	Glu957, Leu959, Glu966	/	/
A6	Aurantiamide Acetate	-7.71	/	Asn1008 (2)[1], Arg1007	/	Lys908
A7	Cerdulatinib	-7.33	/	Glu966, Asp1021, Gly1020	His885	/
A8	CEE321	-7.07	Leu959	Glu966, Asp1021	/	/
A9	Baricitinib	-6.74	/	Glu957, Leu959, Asn1008, Arg1007	/	/
A10	ATI-1777	-6.39	/	Leu959, Glu966	/	/
A11	Momelotinib	-5.30	/	Arg1007, Lys908	/	Arg1007
A12	Brepocitinib	-4.16	/	Glu966, Asn1008, Arg1007	/	/

Note：[1] This indicates that the compound forms two hydrogen bonds with the same amino acid.

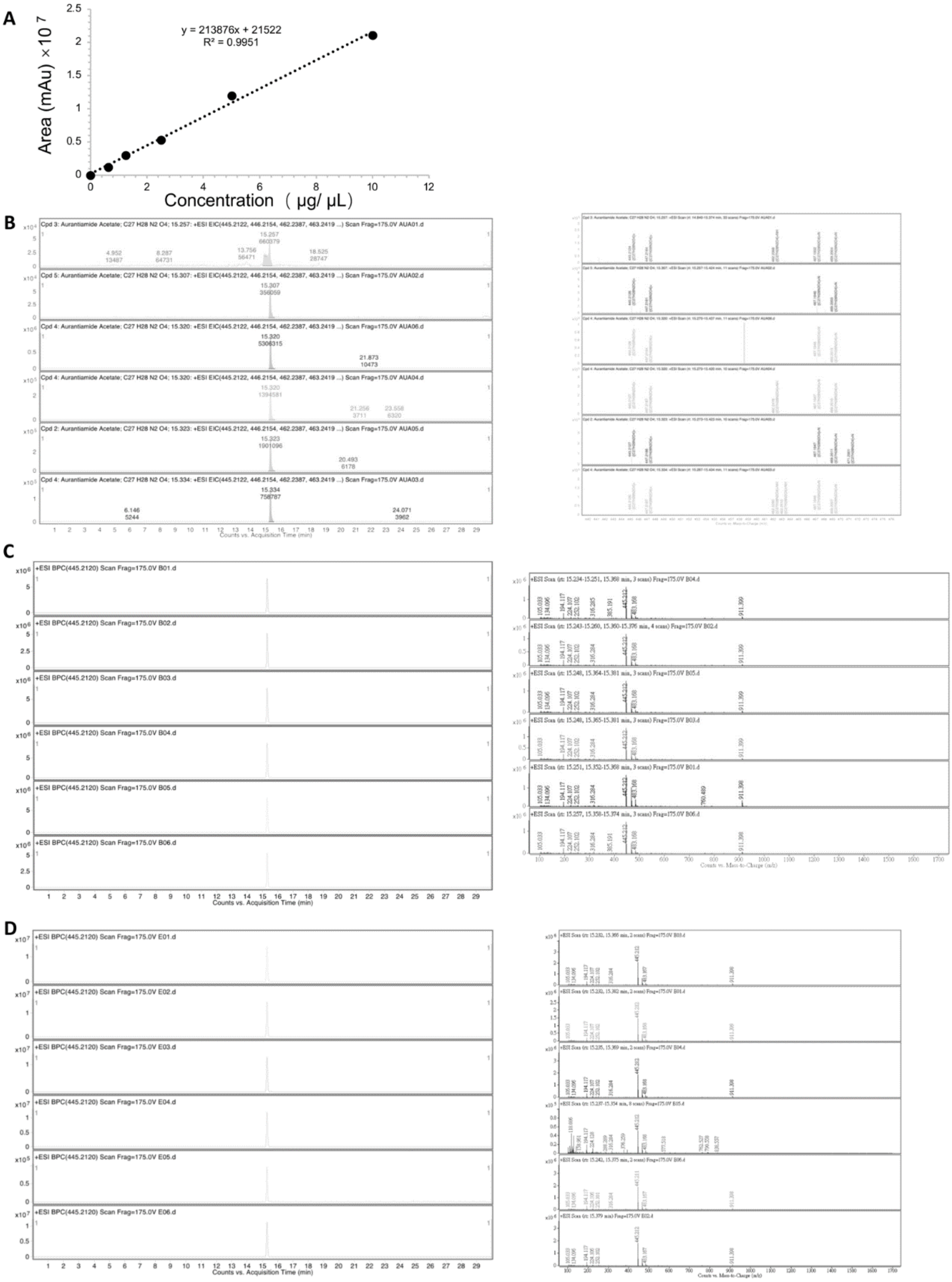

Fig. S1. Chemical analysis of PO aqueous extract. (A) Standard curve of Aurantiamide Acetate established in UHPLC-Q-TOF-MS/MS analyses. (B) Chromatograms (left) and mass spectra (right) for the establishment of a standard curve for Aurantiamide Acetate. (C) Chromatograms (left) and mass spectra (right) of Aurantiamide Acetate detection in the epidermis during transdermal assay. (D) Chromatograms (left) and mass spectra (right) of Aurantiamide Acetate detection in dermis during the transdermal assay.

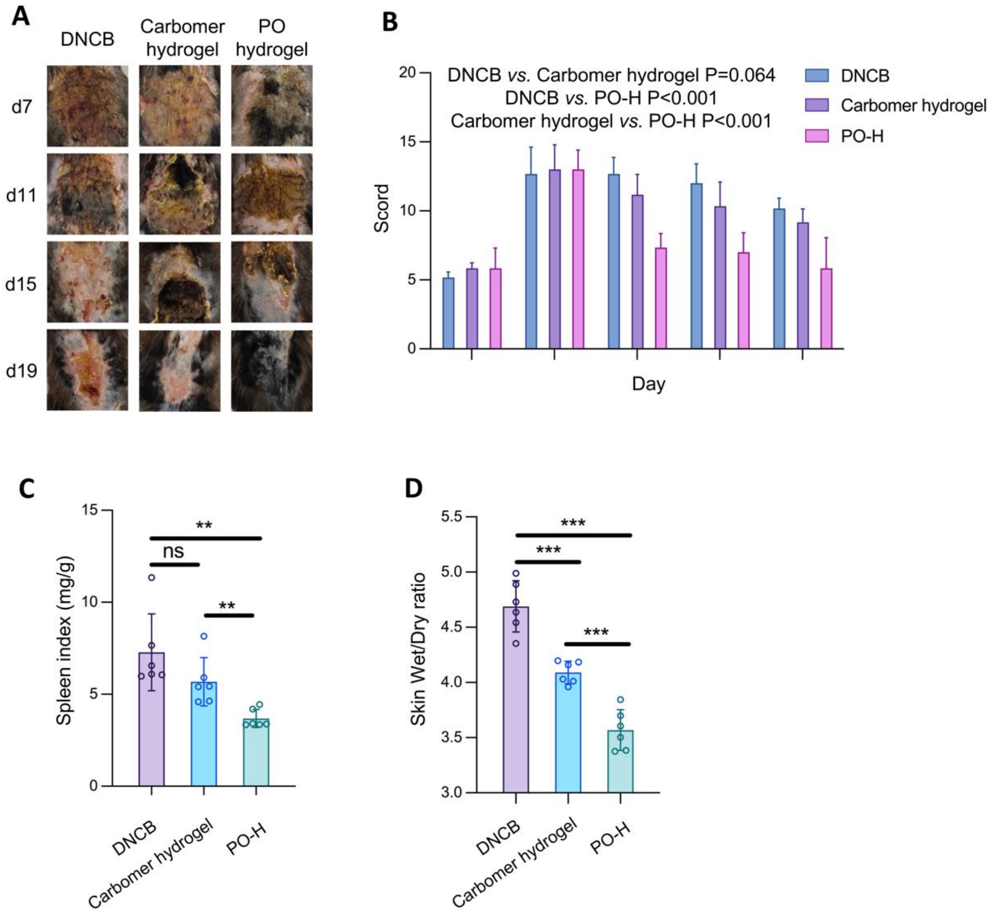

Fig. S2. Comparison between PO hydrogel and carbomer alone on therapeutic effects for DNCB-induced AD symptoms and skin hydration. (A) Representative photographs of the dorsal skin lesions on the 7th, 11th, 15th, and 19th days of the experiment. (B) SCORAD scores were assessed every 4 days. (C) Spleen index of the mice at the end of the study. (D) Dorsal affected skin punches were weighed to calculate wet/dry ratio. The data are expressed as means ± SD, n=6. ** and *** indicate $p < 0.01$ and $p < 0.001$, respectively.

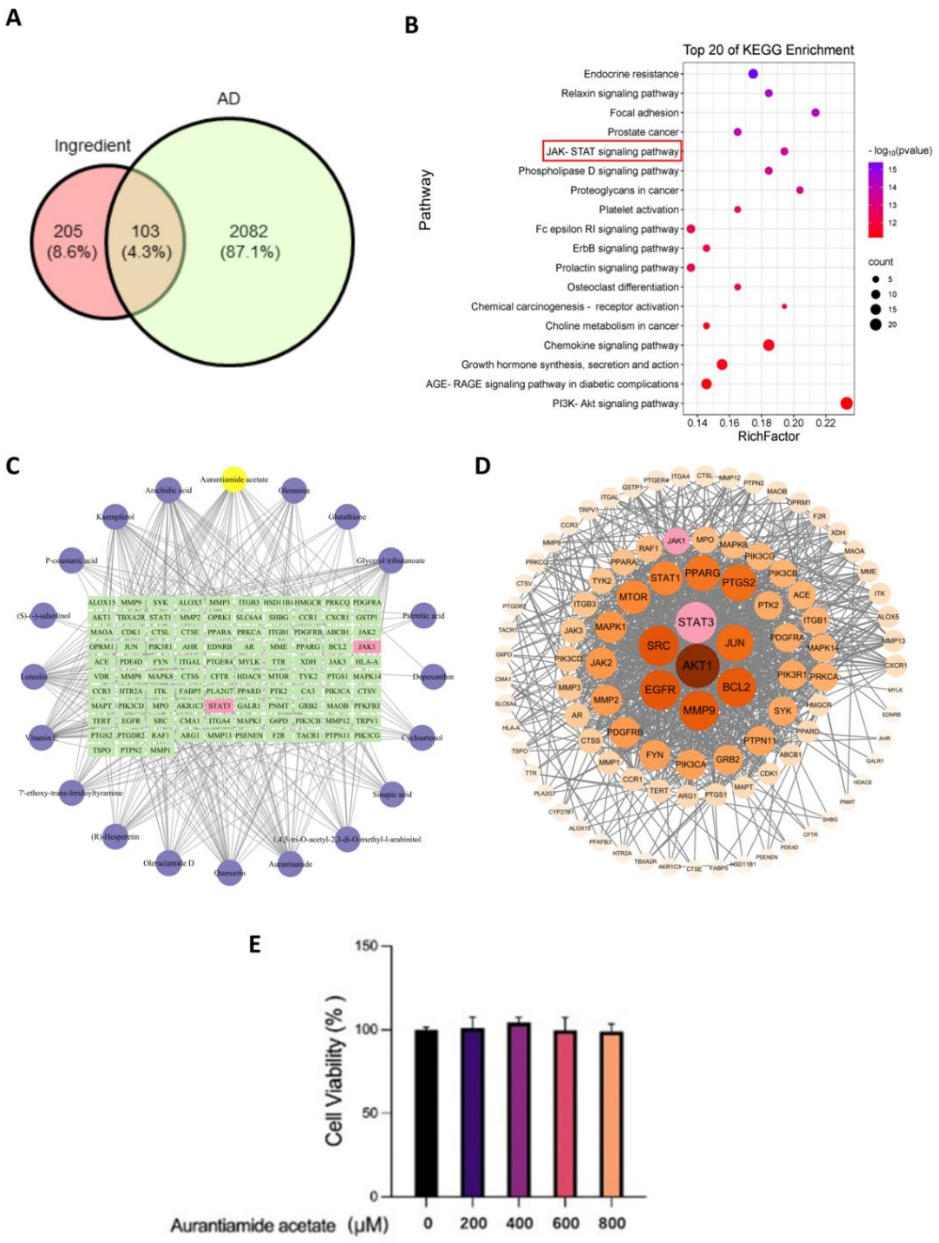

Fig. S3. Network pharmacology analysis of PO. (A) Potential targets of PO ingredients, AD treatment, and the overlapping by Venn diagram. (B) KEGG enrichment analysis of predicted pathways of PO for the treatment of AD. The x-axis represents the number of target genes, and the y-axis represents the –log10 (*p*-value). Colors ranging from red to blue represent *q*-values from small to large. KEGG, Kyoto Encyclopedia of Genes and Genome. (C) The network of PO ingredients and associated targets. Arbitrarily labeled purple and yellow circles represent PO's main and key ingredient, respectively; and arbitrarily labeled green and pink rectangles represent common and key targets of PO in AD treatment. (D) The hub-gene network generated by protein-protein interaction analyses. Each circle represents a protein, and the circle area proportionally represents its relationship with interconnected surroundings. (E) The viability of HaCaT keratinocytes treated with Aurantiamide Acetate or vehicle (0.1% DMSO) for 12h.
